# Subretinal Neovascular Membrane in Wet Age-Related Macular Degeneration Managed With Intravitreal Ranibizumab

**DOI:** 10.7759/cureus.17642

**Published:** 2021-09-01

**Authors:** Tanishq S Sharma, Shashikant M Sharma

**Affiliations:** 1 Medicine, Shree Krishna Hospital, Anand, IND; 2 Ophthalmology, Bhagwati Eye Hospital, Ahmedabad, IND

**Keywords:** wet age-related macular degeneration, ranibizumab, anti-vegf, subretinal fluid, oct

## Abstract

This case report depicts how a case of the subretinal neovascular membrane was managed with intravitreal ranibizumab injections. A 59-year-old female patient presented with complaints of diminution of vision in her right eye for one month. Various necessary examinations were carried out and the patient was diagnosed with both forms of age-related macular degeneration (ARMD) disorder - wet ARMD in the right eye and dry ARMD in the left eye. Pseudophakia was also seen in both eyes. Drusen deposits, characteristic of the disorder, were seen in the macular area of the oculus sinister (OS). The patient was treated for the wet ARMD with intravitreal injections of 0.5 mg ranibizumab administered one month apart in the right eye. The patient showed improvements in her visual acuity and a complete resolution of the subretinal fluid.

## Introduction

Age-related macular degeneration (ARMD) is the leading cause of irreversible blindness among adults over the age of 50 [1]. It is reported to be the third major cause of visual disability worldwide. The disorder manifests in both “wet” and “dry” forms - the former being an advanced stage with exudation of fluid and the latter being an early/intermediate stage of the disorder [2]. Wet ARMD, if left untreated, can lead to permanent loss of central vision. Early diagnosis and management of the condition are crucial to obtain the best results in patients with wet ARMD. The use of anti-vascular endothelial growth factor (VEGF) drugs such as ranibizumab has shown resolution of the subretinal fluid and thus preventing irreversible damage to the retina and improving the visual acuity of the patients. The monthly regimen of intravitreal ranibizumab injections has shown promising outcomes in the management of wet ARMD [3].

## Case presentation

A 59-year-old female patient presented to our ophthalmology outpatient department (OPD) with a complaint of diminution of vision in her right eye for one month. This phenomenon was of sudden and insidious onset with intermittent episodes of hazy vision, eventually leading to the blurring of vision. The patient was a known case of hypertension, dyslipidemia, and hypothyroidism. She was on regular treatment for all these diseases. However, there was no history of diabetes mellitus.

Examination

Ophthalmoscopy and Snellen’s Test 

Initial examination revealed a visual acuity of 6/60 in the oculus dexter (OD) with no improvement with pinhole and the visual acuity in the oculus sinister (OS) was 6/9. Further examination revealed pseudophakia in both eyes.

**Figure 1:**
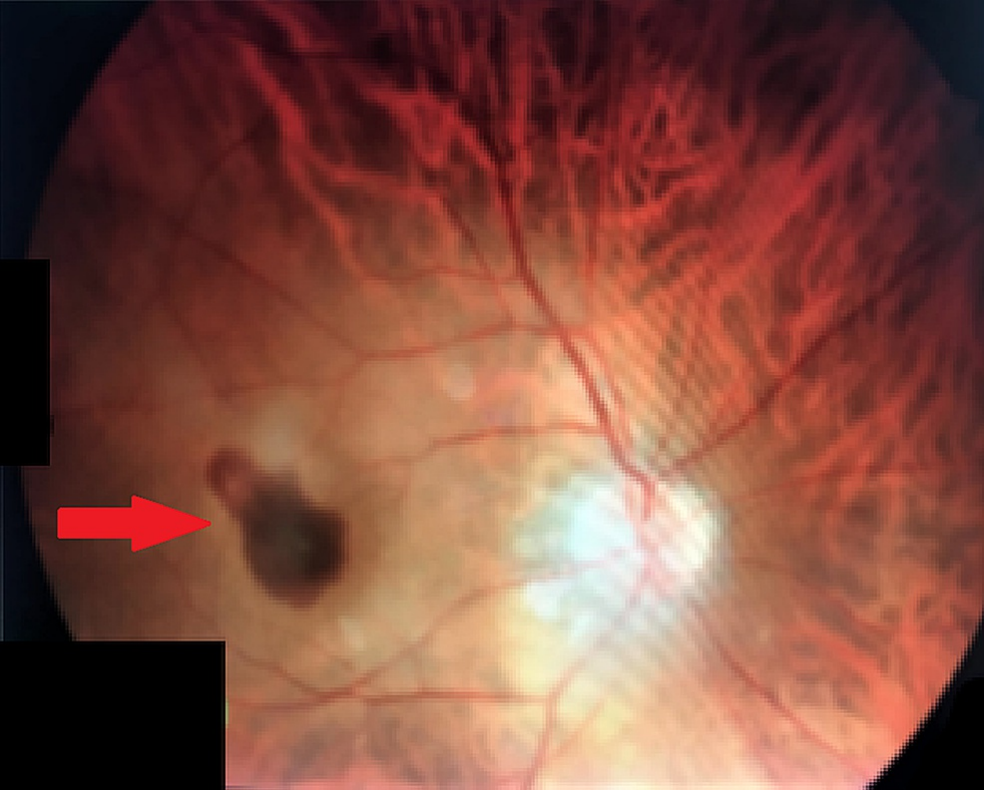
Grey-white SRNVM in OD with subretinal blood and subretinal fluid. SRNVM, subretinal neovascular membrane; OD, oculus dexter.

Fundus examination showed a grey-white membrane in the macular area, also referred to as subretinal neovascular membrane (SRNVM), in the OD with fresh subretinal blood and subtle subretinal fluid (Figure 1). OS showed few drusen in the macular area.

Fundus Fluorescein Angiography 

Fundus fluorescein angiography (FFA) in the right eye confirmed the diagnosis of classic SRNVM.

Optical Coherence Tomography

**Figure 2:**
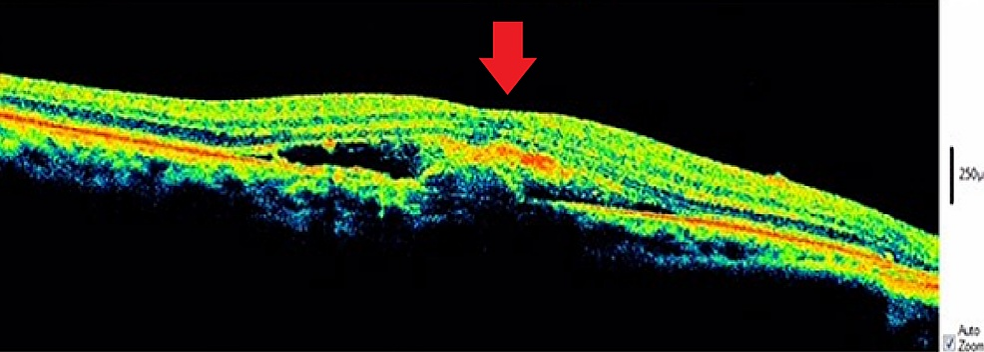
OCT showing macular thickening with active SRNVM complex and accumulation of subretinal fluid accompanied by loss of foveal contour. OCT, optical coherence tomography; SRNVM, subretinal neovascular membrane.

The right eye demonstrated macular thickening with active SRNVM complex, subretinal fluid, and loss of foveal contour (Figure 2).

Diagnosis

Based on the clinical presentation and investigations done, a diagnosis of pseudophakia in both eyes with wet age-related macular degeneration (ARMD) in the right eye and dry ARMD in the left eye was arrived at.

Treatment and follow-up

**Figure 3:**
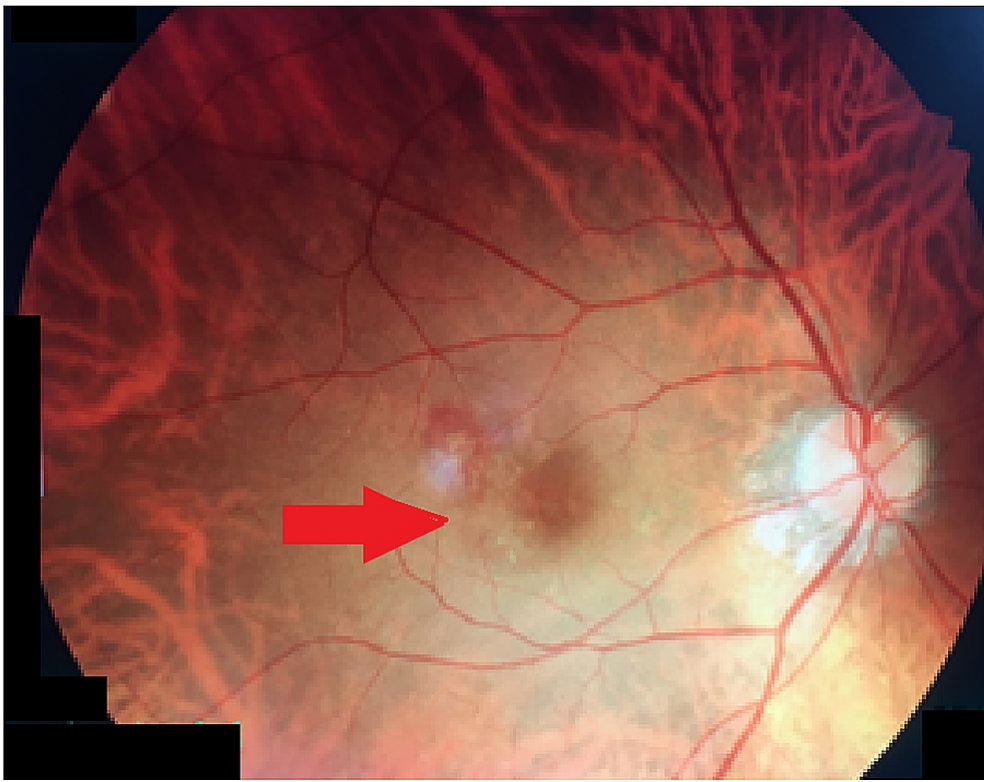
Near-total resolution of subretinal blood with complete resolution of subretinal fluid.

Intravitreal injection of 0.5 mg ranibizumab was administered in the right eye. A follow-up examination carried out after one month revealed an improvement in visual acuity to 6/24 and clinical examination revealed a near-total resolution of the subretinal blood and resolved subretinal fluid (Figure 3).

**Figure 4:**
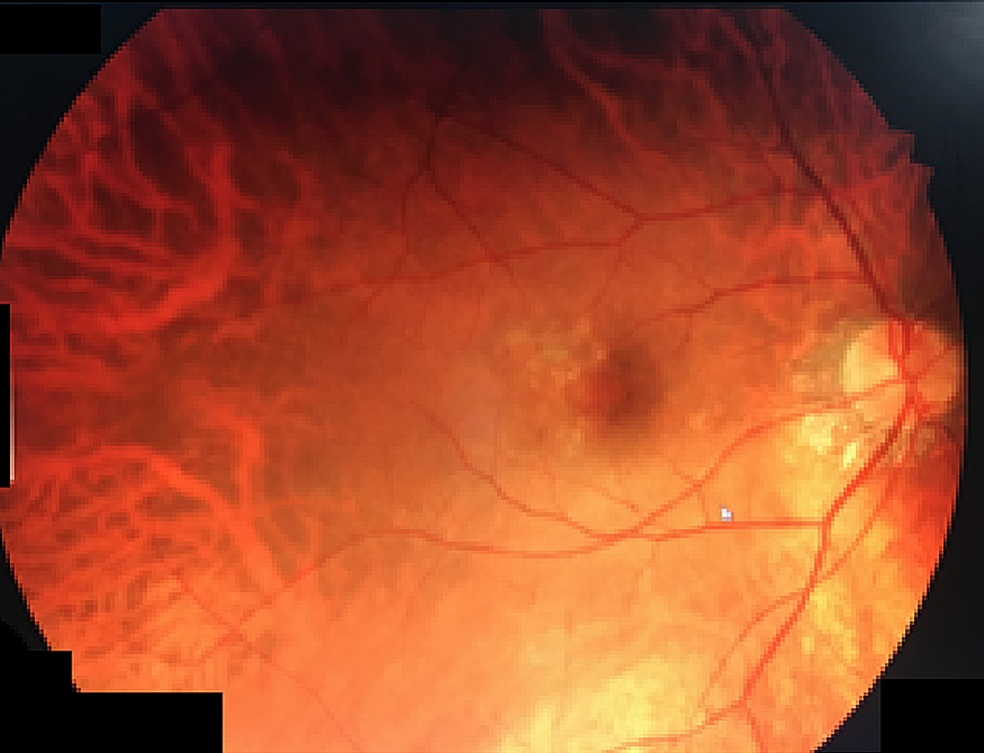
Resolution of SRNVM complex seen as a stained region. SRNVM, subretinal neovascular membrane.

Pro re nata (PRN) treatment protocol was followed. One month after the first dose, the patient was administered a second dose of intravitreal ranibizumab injection 0.5 mg in the right eye. A second follow-up visit revealed a visual acuity of 6/12 and a complete resolution of the subretinal blood and fluid and a dry macula, and the corresponding angiography revealed staining of the resolved SRNVM complex, with no leakage in early or late phases (Figure 4). The patient was administered a total of two doses of intravitreal ranibizumab injection 0.5 mg in the right eye and the follow-up was of three months from her first visit.

**Figure 5:**
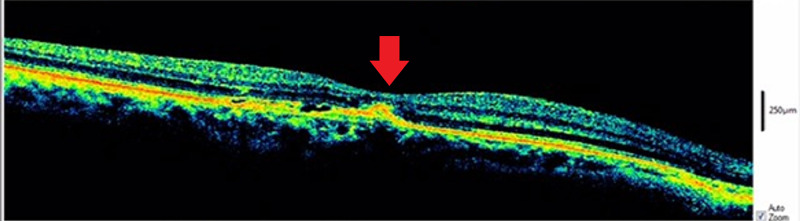
OCT depicting complete resolution of SRNVM and fluid together with the regaining of foveal contour. OCT, optical coherence tomography; SRNVM, subretinal neovascular membrane.

Optical coherence tomography (OCT) of the right eye demonstrated complete resolution of fluid and the SRNVM complex with normal foveal contour regained (Figure 5).

## Discussion

Age-related macular degeneration (ARMD) is the leading cause of irreversible blindness among adults over the age of 50 [1]. It is a neurodegenerative disease that results in progressive loss of vision. The global prevalence of ARMD is about 170 million individuals, while in Asia, it has been recorded as 7.4% and it is reported to be the third major cause of visual disability worldwide. Several genetic and environmental factors are known to precipitate the etiology of the disorder although the precise pathogenesis mechanisms are inconclusive despite several hypotheses that have been proposed for the same. The disorder manifests in both “wet” and “dry” forms - the former being an advanced stage with exudation of fluid and the latter being an early/intermediate stage of the disorder. Drusen deposits, the principal characteristic of the disorder, are known to accumulate as a consequence of the influx of aberrant lipids into the retinal pigment epithelium and their efflux from the same [4].

The patient, in the present case scenario, presented with diminution of vision in her right eye for a month, which was of sudden and insidious onset, commencing with intermittent episodes of hazy vision eventually leading to diminished vision. The patient was diagnosed with both forms of the disorder - wet ARMD in the right eye and dry ARMD in the left eye. Pseudophakia was also seen in both eyes. Drusen deposits, characteristic of the disorder, were seen in the macular area of the OS.

The wet ARMD in the right eye was managed using 0.5 mg ranibizumab intravitreal injection. Several landmark studies have endorsed the utility of ranibizumab in the management of ARMD. The minimally classic/occult trial of the anti-VEGF antibody ranibizumab in the treatment of neovascular age-related macular degeneration (MARINA) study demonstrated a significant improvement in visual acuity (p < 0.001) with ranibizumab over sham-treatment. The anti-VEGF antibody for the treatment of predominantly classic choroidal neovascularization in age-related macular degeneration (ANCHOR) study in predominantly SRNVM patients demonstrated similar significant results in terms of the efficacy of ranibizumab over photodynamic therapy (PDT) (p < 0.0001) [5].

Garcia-Layana et al. [6] reviewed and concluded that ranibizumab was effective in the therapeutic management of wet ARMD. They also summarized the potential benefits of proactive treatment with ranibizumab, which include:

(1) Anticipating relapses or recurrences and, therefore, avoiding drops in vision while individualizing patient follow-up.

(2) Avoiding irreversible loss of vision at recurrences despite reactive treatment.

(3) Reduction in the number of injections and their associated risks.

(4) Reduction in the number of visits and their associated costs and inconvenience.

Recently published Greek guidelines also support the net benefit accrued upon monthly injections of ranibizumab in the event of a confirmed diagnosis of choroidal neovascularization due to wet ARMD [7].

## Conclusions

Early diagnosis and proper management of wet ARMD can improve the vision of the patient. In this case, the patient responded well to ranibizumab therapy, as seen from a steady improvement in visual acuity, resolution of subretinal blood and subretinal fluid, and eventual progressive drying of the macula without fluid leakage, as seen from angiographic and OCT findings at different stages of follow-up. From the available literature cited herein, it can be concluded that treatment with ranibizumab was appropriate in the present circumstances.
